# Use of the International League Against Epilepsy (ILAE) 1989, 2010, and 2017 Classification of Epilepsy in children in a low‐resource setting: A hospital‐based cross‐sectional study

**DOI:** 10.1002/epi4.12401

**Published:** 2020-06-21

**Authors:** Suvasini Sharma, Aakanksha Anand, Divyani Garg, Sakshi Batra, Sharmila B. Mukherjee, Bijoy Patra, Satinder Aneja

**Affiliations:** ^1^ Department of Pediatrics Lady Hardinge Medical College and associated Kalawati Saran Children’s Hospital New Delhi India; ^2^ Department of Neurology Lady Hardinge Medical College and associated Smt. Sucheta Kriplani Hospital New Delhi India

**Keywords:** classification, epilepsy, ILAE, seizure

## Abstract

**Objectives:**

This cross‐sectional study was designed to test the applicability of the 1989, 2010, and 2017 International League Against Epilepsy (ILAE) classification of epilepsy in children from a resource‐limited setting in India.

**Methods:**

Classification of seizure types and syndromes was done through parental interviews and review of medical records in children with epilepsy aged one month to 18 years. Available investigations including EEG, MRI, and metabolic/genetic tests were used in classifying patients as per the 1989, 2010, and 2017 ILAE (level II‐epilepsy type) classification. We compared the proportion of children remaining unclassified by each scheme.

**Results:**

Seven hundred and twenty‐six children (436 males, mean age 6.4 ± 4.6 years) were enrolled. Using the 1989 ILAE classification, we were able to classify 95.7%, and 82.6% children by the 2010 scheme. The 2017 ILAE classification could classify all 726 children at level I (seizure type), 664 (91.0%) children at level II (epilepsy type), and an electroclinical syndrome could be identified in 409 (56.1%) of the children. An etiology could be identified in 75%, perinatal brain injury being the most frequent. West syndrome was the most common electroclinical syndrome, identified in 22.7% patients. The 1989 ILAE classification system was superior to the 2010 system (*P* = .01) in epilepsy classification. There was no difference between the 1989 and 2017 schemes (*P* = .31) or the 2010 and 2017 schemes (*P* = .10).

**Significance:**

The 2017 ILAE classification, being multidimensional, allowed classification of children who could not undergo extensive evaluation due to economic constraints and also provided room for overlapping etiologies.

**Key points**

- Most children with epilepsy could be classified in terms of epilepsy type using the 1989 and 2017 ILAE schemes than the 2010 scheme.
- The 1989 scheme was superior to the 2010 scheme in classification but equivalent to the 2017 scheme. The 2017 and 2010 ILAE schemes were equivalent for epilepsy classification.
- The 2017 classification scheme allows multidimensional classification even in a resource‐limited setting.

## 1 INTRODUCTION

Around 70 million people are living with epilepsy worldwide, with 80% of them residing in low‐ and middle‐income countries.^1^ An estimated 12 million people with epilepsy live in India, contributing to almost one‐sixth of the global burden of disease.^2^ In low‐resource countries such as India, significant treatment gaps have been reported to exist in epilepsy.^3^ Treatment gap is defined as the number of people with active epilepsy not on treatment or on inadequate treatment expressed as a percentage of total number of people with active epilepsy.^3^

The primary aim of epilepsy classification is to provide a common international language and terminology for clinical practice. Classification also establishes a framework for understanding and formulating research. Classification of epilepsy is complex in children, due to extremely variable clinical presentation with multiplicity of seizure types and high prevalence of epilepsy syndromes. Additionally, presence of perinatal, genetic, and metabolic causes in childhood epilepsy necessitates access to advanced and expensive diagnostics in order to conduct the classification.

Epilepsy classification, since its inception, has been fraught with complexity and often controversy. The first classification system of seizures by the International League Against Epilepsy (ILAE) appeared in 1969/1970 which classified seizures into partial and generalized forms.^4^ The ILAE introduced a new classification in 1985 into four categories based on the two axes: idiopathic/symptomatic epilepsy and generalized/ localized epilepsy which incorporated seizure semiology, EEG, age at presentation as well as seizure frequency.^5^ The concept of cryptogenic epilepsies was introduced in 1989.^6^ In 2001, a diagnostic scheme consisting of five axes describing the semiology (axis 1), seizure type (axis 2), syndrome (axis 3), etiology (axis 4) and disability (axis 5) was proposed by the ILAE Task Force,^7^ followed by a revised classification in 2010, which recognized advances in neuroimaging, genomic technologies and molecular biology which needed to be incorporated in a fresh scheme.^8^ The basic dichotomy into focal and generalized epilepsy was unlikely to support information obtained from neuroimaging, genetic and molecular studies, and hence, new groups were introduced that included “electroclinical syndromes”, “distinct constellations,” “epilepsies attributable to structural‐metabolic cause,” and “epilepsies of unknown cause.” The advantages of this system included explicitly defined categories. However, the classification was dependent on investigations for clear categorization.

After variable feedback to the 2010 scheme worldwide, another classification system was proposed in 2017 which consisted of classifying epilepsy at various “levels” such as type of seizure, type of epilepsy, and whether an electroclinical syndrome could be defined.^9^ An etiological factor was sought for at all levels which included immune and infectious causes in addition to the previous genetic, structural, and metabolic causes, with room for further expansion. The 2017 system allows enhanced precision of epilepsy diagnosis as well as classification at multiple levels depending on the information and resources available, and seems to permit more inclusive categorization. However, it removes the broad syndromic categorization offered by the 1989 classification system.

Evaluation of these various classification schemes, particularly in pediatric epilepsy, is scarce. For appropriate applicability of the 2017 classification, electroencephalography (EEG), preferably ictal video EEG to document the seizure onset, neuroimaging, preferably MRI, and genetic and metabolic testing are needed. These modalities are not frequently available in low‐ and middle‐income countries.

However, whether the 2017 paradigm is truly beneficial clinically and serves greater utility compared to previous systems in classifying patients, specifically children with epilepsy, has been evaluated in only one large‐scale study, but never in resource‐limited regions.^10^ We therefore sought to test the utility of each of the ILAE classification systems (1989, 2010, and 2017) in a resource‐limited setting and also describe the spectrum and etiology of childhood epilepsy in our setting.

## 2 METHODS

### 2.1 Study setting and population

This study was conducted in the Epilepsy Clinic of Kalawati Saran Children's Hospital, a government‐funded tertiary referral center in New Delhi, India. This is a once‐weekly clinic, wherein 100 to 150 children with epilepsy are seen every week. Ours is a tertiary care government‐sponsored teaching hospital which caters to Delhi and surrounding districts of Haryana, Uttar Pradesh, and Punjab. Our patients predominantly belong to lower socioeconomic status and do not have any form of health insurance. Being a tertiary‐level center, we have a higher proportion of complicated referral cases although we do have fair proportions of non‐referred cases as well. The Epilepsy Clinic caters to an admixture of drug refractory as well as pharmacoresponsive epilepsy cases.

The study was conducted from May 2017 to October 2018. Institutional Ethical Committee approval was obtained. Written informed consent was taken from the parents.

Children of age one month to 18 years fulfilling the 2014 ILAE operational definition of epilepsy were enrolled. As per this definition, epilepsy is defined as

1. At least two unprovoked (or reflex) seizures occurring greater than 24 hours apart.
2. One unprovoked (or reflex) seizure and a probability of further seizures similar to the general recurrence risk (at least 60%) after two unprovoked seizures, occurring over the next 10 years.
3. Diagnosis of an epilepsy syndrome

Children with febrile seizures and acute symptomatic seizures, including those presenting with seizures caused by ring‐enhancing lesions, that is, inflammatory granulomas, were excluded. We did however include children who had epilepsy caused by calcified granulomas. Consecutive children presenting to the clinic were enrolled. These included both newly diagnosed as well as follow‐up patients.

Systematized history and examination were conducted for each patient and entered into a predesigned data record form. The results of investigations performed were noted. As per our clinic protocol, all patients with epilepsy undergo short‐term video EEG. Home videos were also reviewed whenever available. Neuroimaging was done in all cases except in genetic generalized epilepsy. Although magnetic resonance imaging (MRI) of the brain is the investigative modality of choice for evaluation of epilepsy, patients often underwent Computed Tomography (CT) scan due to financial constraints. Genetic studies were performed if the electroclinical characteristics were strongly suggestive of a genetic cause/syndrome, and parents could afford the testing. Tandem mass spectrometry (TMS) and gas chromatography‐mass spectrometry (GC‐MS) were done in cases of unexplained epileptic encephalopathy or when the child had other features suggestive of inborn errors of metabolism.

All the patients underwent a short‐term video EEG of 30‐60 minutes duration. A total of 689 (95%) children underwent CT scan, while 576 (79%) underwent MRI. 463 (64%) had undergone initial CT, followed by MRI as CT was non‐diagnostic. 153 (21%) children underwent metabolic screen‐ blood gas, lactate, plasma amino acid and organic acid profile, and urine GC‐MS. Forty‐four children underwent genetic testing; 3 children underwent targeted genetic testing (2—SCN1A, 1—CDKL5). Eight children underwent next generation sequencing—clinical exome testing.

### 2.2 Classification of epilepsy

Epileptic seizures and type of epilepsy as well as etiology of epilepsy were classified through parental interviews and review of medical records, using standardized data collection forms. All investigations available including EEG, MRI, and metabolic and genetic tests were used in classifying patients as per the 1989, 2010, and 2017 ILAE classification. The classification was done by the pediatric neurologists (SS and SA) involved in the study.

### 2.3 Outcomes

The primary outcome measure was the proportion of patients who could be classified using the three classification systems. Secondary outcomes included the spectrum of seizure types and epilepsy syndromes, etiology, and comorbidities.

### 2.4 Statistical analysis

Statistical analysis of the data was performed using IBM SPSS software (IBM SPSS Statistics for Windows, Version 22.0. Armonk, NY: IBM Corp.). Categorical data were expressed as frequency and percentage. Continuously distributed data were expressed as mean ± SD. We divided children with epilepsy by type of seizure, epilepsy and etiology, and calculated proportions within the study population. We compared the various classification systems using the McNemar chi‐square test. A p value of less than 0.05 was considered statistically significant.

## 3 RESULTS

### 3.1 Demographic profile and seizure types

A total of 726 children (436 males) with epilepsy were enrolled into the study. The mean age at presentation was 6.4 years (SD 4.6; Table 1). Age at onset ranged from one month to 16 years, with a mean age at onset of 4.6 years (SD 3.9). 63% of children presented in the childhood years (between 1 and 10 years), 23% during adolescence, and 14% in infancy (1‐12 months). According to the 2017 classification of epileptic seizures, 351 children (48.3%) had focal onset seizures and 201 (27.6%) had generalized onset seizures (Table 2). In 143 children (19.6%), the seizure onset was unknown, and in 31 children (4.2%), the seizure type could not be classified.

**TABLE 1:** Characteristics of study participants

Characteristics	N = 726 n (%)
Male gender	436 (61.5%)
Mean age at presentation (y) ±SD	6.4 ± 4.6
Mean age at onset (y) ±SD	4.6 ± 3.9
Onset	
Infancy	104 (14%)
Childhood	455 (63%)
Adolescence	167 (23%)
Family history of epilepsy	115 (16%)
History of febrile seizures	76 (10%)
>1 seizure type reported	110 (15%)
Comorbidities	
Cerebral palsy	164 (23%)
Developmental Delay/Intellectual disability	349 (11%)
Autistic features	42 (6%)
Behavioral issues	53 (7%)
Neuroregression	29 (4%)
Feeding difficulty	57 (8%)
Poor school performance	85 (12%)
Vision impairment (including refractory errors and squint)	157 (22%)
Hearing impairment	44 (6%)
Hyperactivity	65 (9%)
Sleep problems	31 (4%)

Abbreviation: SD, standard deviation

**TABLE 2:** Classification of seizures as per 2017 ILAE seizure classification

ILAE 2017 Seizure Classification	Total N = 726^a^ n (%)
Focal onset	351(48%)
Awareness	
Focal onset aware	24
Focal onset with impaired awareness	327
Motor vs Non‐motor onset	
Motor onset	189
Non‐motor onset	126
Focal to Bilateral tonic‐clonic	156
Generalized onset seizures	201 (28%)
Motor	136
Tonic‐clonic	32
Clonic	2
Tonic	23
Myoclonic	34
Atonic	16
Myoclonic atonic	3
Myoclonic tonic‐clonic	11
Epileptic spasms	15
Non‐motor (absences)	65
Typical absences	32
Atypical absences	28
Other absences (myoclonic, eyelid myoclonia, others)	5
Unknown onset	143 (20%)
Motor	27
Tonic‐clonic not classifiable	92
Epileptic spasms	24
Non‐motor behavioral arrest	
Unclassified	31 (4%)

^a^ In patients with > 1 seizure type, the predominant seizure type was included for classification.

### 3.2 Electroclinical syndromes

West syndrome was found to be the most common epilepsy occurring in our setup. Benign childhood epilepsy with centrotemporal spikes (BECTS) was the most common among the focal epilepsies. Genetic generalized epilepsies, earlier termed idiopathic generalized epilepsies (IGE), constituted the largest subset of generalized epilepsy. (Table 3).

**TABLE 3:** Electroclinical syndromes in the study population (n‐726)

Electroclinical Syndrome (N = 726)	n (%)
West syndrome	165 (23%)
Benign epilepsy with centrotemporal spikes	42 (6%)
Epilepsy with Febrile seizure plus	28 (4%)
Juvenile myoclonic epilepsy	25 (3%)
Lennox‐Gastaut syndrome	23 (3%)
Epilepsy with GTCS alone	21 (3%)
Childhood absence epilepsy	18 (3%)
CSWS‐LKS spectrum	17 (3%)
Panayiotopoulos syndrome	15 (2%)
Epilepsy with myoclonic atonic seizures	11 (2%)
Juvenile absence epilepsy	11 (2%)
Autosomal dominant nocturnal frontal lobe epilepsy	9 (1%)
Dravet syndrome	8 (1%)
Reflex Epilepsies	4 (0.5%)
Late onset occipital lobe epilepsy	3 (0.4%)
Epilepsy with myoclonic absences	3 (0.4%)
Ohtahara syndrome	3 (0.4%)
Early myoclonic encephalopathy	2 (0.3%)
Epilepsy of infancy with migrating focal seizures	1 (0.1%)
Classified	409 (56%)
Unclassified	317 (44%)
Total	726 (100%)

Abbreviations: CSWS, continuous spike waves during sleep; GTCS, generalized tonic‐clonic convulsions; LKS, Landau‐Kleffner syndrome.

### 3.3 Etiology

The etiology of epilepsy could be identified in 75% of the children (Table 4). Perinatal brain injury was the commonest cause, seen in 265 children (36.5%). The perinatal causes included asphyxia, hypoglycemia, sepsis/meningitis, intracranial hemorrhage, and stroke. Genetic causes were the next most common category, seen in 149 children (20.5%). These predominantly included epilepsy syndromes with a presumed genetic basis (105 children, 14.4%); such as benign childhood epilepsy with centrotemporal spikes, childhood absence epilepsy, juvenile myoclonic epilepsy, juvenile absence epilepsy, epilepsy with febrile seizure plus, epilepsy with myoclonic atonic seizures, and autosomal dominant frontal lobe epilepsy. Other genetic causes included diagnosed single gene disorders caused by epilepsy associated genes (11), inborn errors of metabolism (7), genetic malformations of cortical development (11), and neurocutaneous disorders (15).

**TABLE 4:** Etiology of Epilepsy in study population

Etiology of epilepsy	N (%) (Total n = 726)
Perinatal brain injury	265 (37%)
Asphyxia	129 (18%)
Symptomatic hypoglycemia	103 (14%)
Sepsis/meningitis	26 (4%)
Intracranial hemorrhage	5 (0.6%)
Stroke	2 (0.2%)
Postnatal brain injury	124 (17)
Calcified granulomas	71 (10%)
Meningoencephalitis	37 (5%)
Trauma	5 (0.6%)
Mesial temporal sclerosis	5 (0.6%)
Tumors^a^	4 (0.5%)
Rasmussen's encephalitis	2 (0.2%)
Hypoxic brain injury	2 (0.2%)
Acute disseminated encephalomyelitis	1 (0.1%)
Stroke	1 (0.1%)
Hemiplegia hemiconvulsion epilepsy	1 (0.1%)
Prenatal causes	158 (22%)
Genetic	149 (21%)
Diagnosed single gene disorders^b^	11(2%)
Epilepsy syndromes with presumed genetic etiology^c^	105 (14%)
Inborn errors of metabolism^d^	7 (1%)
Neurocutaneous disorders^e^	15 (2%)
Presumed genetic malformations of cortical development^f^	11 (2%)
Focal cortical dysplasias	5 (0.6%)
Intrauterine infections	4 (0.5%)
Unknown	179 (25%)

^a^ Hypothalamic hamartomas (2), Dysembryoplastic neuroepilethelial tumors (2)

^b^ SCN1A mutations (8), KCNT1 mutation (1), CDKL5 mutation (1), and STXBP1 mutation(1)

^c^ Juvenile myoclonic epilepsy (25), Juvenile absence epilepsy (11), Childhood absence epilepsy (18), Benign childhood epilepsy with centrotemporal spikes (42), Epilepsy with febrile seizure plus (28), Epilepsy with myoclonic atonic seizures (11), and Autosomal dominant nocturnal frontal lobe epilepsy (9)

^d^ Phenylketonuria (2), Glutaric aciduria (2), Methylmalonic academia (1), Pyridoxine dependency (1), and Biotinidase deficiency (1)

^e^ Tuberous sclerosis (12), Sturge‐Weber syndrome (2), and Hypomelanosis of Ito (1)

^f^ Lissencephaly (8), Bilateral frontoparietal polymicrogyria (2), and Subcortical band heterotopia (1)

Acquired infections in children were also found to be an important cause of epilepsy, with calcified granulomas in 71 children (9.7%) and sequelae of meningoencephalitis in 37 children (5%). Five children had epilepsy as a sequelae of head trauma, and four children had epilepsy secondary to brain tumors.

We also classified the etiology subtypes as per the ILAE 2017 classification (Table 5). Structural causes were the most predominant, seen in 431 children (59.3%), followed by genetic (149 children, 20%) and infectious (134 children, 18.4%) causes. There was a considerable overlap between the categories. For example, tuberous sclerosis was classified as both structural and genetic, epilepsy caused by meningoencephalitis sequelae was classified as both structural and infections, and epilepsy caused by genetic malformation was classified as both structural and genetic.

**TABLE 5:** Etiological subtypes as per the ILAE 2017 Classification^a^

Etiology	N (%) Total n = 726
Structural	431 (59%)
Genetic	149 (21%)
Metabolic	7 (1%)
Infectious	134 (18%)
Immune	3 (0.4%)
Unknown	179 (25%)

^a^ Overlaps between categories common

### 3.4 Use of the ILAE classifications of epilepsy

#### 3.4.1 1989 ILAE Classification of Epilepsy

Six hundred and ninety‐five children (95.7%) of our cases could be classified on applying the 1989 classification system (Table 6). Three hundred and fifty‐one children (48.3%) patients had “localization related epilepsies and syndromes,” and 201 (27.6%) had “generalised epilepsies and syndromes.” “Epilepsies undetermined epilepsy, whether focal or generalised” was seen in 56 children (7.7%) of the patients. Undetermined unequivocal focal or generalized epilepsy was seen in 83 patients (11.4%). We faced problems with classifying certain epilepsy syndromes such as generalized epilepsy with febrile seizure plus, as this syndrome was not recognized in this classification. We classified it as generalized, idiopathic. Also, febrile seizures have been classified in this scheme into special syndromes although we excluded febrile seizures from our study, as they do not constitute epilepsy.

**TABLE 6:** Classification of epilepsy as per the 1989, 2010, and 2017 ILAE classification

Classification of Epilepsy—1989 Classification (N=726)			
	Number of patients		Proportion of total study population n=726
Localization related (Total n=351)		n=351	
Idiopathic	94	26.9%	13%
Symptomatic	181	51.5%	25%
Cryptogenic	76	21.6%	10%
Generalized (Total n=201)		n = 201	
Idiopathic	116	57.7%	16%
Symptomatic	56	27.8%	8%
Cryptogenic	29	14.4%	4%
Undetermined—both focal and generalized	56		8%
Undetermined—unequivocal focal/generalized	83		11%
Special situations	4		0.4%
Not classified	31		4%

Classification of Epilepsy—2010 Classification (N=726)		
Type	n	% N
Electroclinical syndrome	409	56%
Distinct constellation	10	1%
Structural‐metabolic cause (and not categorized into epilepsy syndrome or constellation)	181	25%
Unknown cause (and not classified into any of the above categories)	126	17%

Classification of Epilepsy—2017 Classification (N=726)		
Type	n	% N
Seizure type—level 1	726	100%
Epilepsy type—level 2	664	91%
Syndrome—level 3	409	56%
Etiology—level 4	544	75%
Co morbidity—level 5	488	67%

#### 3.4.2 2010 ILAE Classification of Epilepsy

Six hundred (82.6%) of the patients could be classified by the 2010 scheme (Table 6). Four hundred and nine (56.1%) of the children had “electroclinical syndromes.” “Distinct constellation” was identified in ten children (1.3%). One hundred and eighty‐one children (24.9%) had epilepsy caused by structural‐metabolic causes which could not be classified into epilepsy syndrome or constellation. One hundred and twenty‐six children (17.3%) had epilepsy of unknown cause which could not be classified in any of the above‐mentioned categories.

#### 3.4.3 2017 ILAE Classification of Epilepsy

The 2017 ILAE classification could classify all 726 children at level I (seizure type), 664 (91%) children at level II (epilepsy type), and an electroclinical syndrome could be identified in 409 (56.1%) of the children. An etiology could be identified in 75% of the cases by this classification, and comorbidities were identified in 67% of the children with epilepsy (Table 6).

The three classification systems were compared using the McNemar chi‐square test using the proportion of patients who could not be classified. The 1989 scheme was superior to the 2010 scheme (*P* = .01). There was no statistically significant difference between the 1989 and 2017 classification systems (*P* = .31) or the 2010 and 2017 schemes (*P* = .10).

## 4 DISCUSSION

In the present study, we attempted to classify a large group of children with epilepsy in a low‐resource setting with each of the three ILAE classification systems (1989, 2010, and 2017). Using the 1989 classification scheme, 94.9% of our patients could be classified. These results are consistent with previous studies in adults, with the success of classification ranging from 85.5% to 100%.^11^, ^12^, ^13^, ^14^ A Norwegian study in the pediatric population was able to assign a broad syndromic classification to 93% of epilepsies using this scheme.^10^

The 2010 classification scheme enabled categorization of 82.6% of patients in our study. This classification scheme seems to be less successful than the 1989 classification. In a previous study conducted in India, 84% of adult patients with epilepsy could be classified using this scheme.^15^ This could be because the 2010 scheme was envisaged to incorporate advances in genetic and molecular diagnosis as well as progress in neuroimaging. However, in a resource‐limited setting like ours, advanced genetic testing and neuroimaging are often determined by financial constraints. Additionally, even if genetic testing is available, the genetic structure of most idiopathic epilepsies remains unclear. That genetics should form a basis for classification in the 2010 classification hence seems impractical. The 2010 scheme also replaces “symptomatic epilepsies” with “Epilepsies attributed to and organized by structural‐metabolic cause.” However, there are several epilepsies that arise from neither a structural nor a metabolic cause such as autoimmune or toxin‐induced that may not find their true place within this category. However, the 2010 classification was simple to administer and also had provisions to leave epileptic spasms as “unknown onset,” as these could also arise from a focal etiology.

The 2017 classification successfully classified 91% of epilepsies leading to 9% of epilepsies remaining unclassified. In a similar study in Indian children testing the applicability of the 1981/1989/2017 systems, seven percentage of children with epilepsy remained unclassified using the 2017 scheme.^10^

As per the ILAE 2017 classification, 24.7% of our patients had unknown cause of epilepsy. The remaining majority could have an etiological categorization. This contrasts with the data in previous studies wherein the proportion of patients with a determinable cause varied from 18% to 33%.^16^, ^17^, ^18^ In the study by Aaberg et al, 67% of epilepsy belonged to the “unknown” category in the 1989 and 43% in the 2017 classification,^10^ proportions being higher than our population. This could be because central nervous system infections such as neurocysticercosis constitute a significant bulk of epilepsy in our setting and are demonstrable with ease even on non‐contrast neuroimaging, enhancing detection rates.^19^ Fifteen percentage of etiological categorization in our study was borne by infections. Another contributory factor was the occurrence of perinatal insults (seen in 32.4% of our patients). Structural changes induced in the brain due to perinatal insult strongly enhanced the structural etiological category. Perinatal insult was determined to contribute to the development of 50% of pediatric epilepsy under the age of three years in a hospital‐based study in India.^20^, ^21^ Only 16% of our patients could be demonstrated to have a genetic cause in comparison with data from developed countries as testing could not be offered to all patients. Another observation in our study was that proportions of children with benign and self‐limiting epilepsies such as BECTS, CAE, and Panayiotopoulos syndrome were low compared with the Norwegian study by Aaberg et al This difference could partly result from the much higher prevalence of perinatal and postnatal brain injuries and infections in our study population. Another reason could be that children with benign and self‐limited epilepsies were less likely to be referred to our tertiary‐level referral clinic.

Considering that epilepsy has a complex multifactorial origin, Berg et al proposed that the order and organization of the list of recognized syndromes need not be singular, constrained, or rigid but should be flexible to reflect our best current understanding.^22^ This flexibility led to considerable overlap between the etiologies and syndromic entities in the 2010 ILAE classification. For example, cortical malformations and neurocutaneous syndromes both have a proven genetic basis, and therefore, the etiology of epilepsy can be both genetic and structural. There are various epilepsies such as West syndrome, Lennox‐Gastaut syndrome, and CSWS which are classified as electroclinical syndromes but majority of them have a known structural—metabolic etiology.^23^, ^24^ Genetic associations for West syndrome have also been described (STXBP1, ARX homeobox mutation).^25^ Also, patients with the same electroclinical syndrome do not always share the same underlying cause (West syndrome, Lennox‐Gastaut syndrome, and epilepsy with CSWS), and the same etiologic factor may be associated with a range of epilepsy phenotype expression; for example, SCN1A mutations lead to GEFS + and Dravet syndrome.^26^

The 2017 ILAE classification system allows classification at multiple levels and gives a prospect to avoid overlaps, avoid reclassification when there is evolution of one syndrome to another, and identify multiple etiologies when present but it is not without its shortcomings. A lot of emphasis is laid on the onset of seizures and epilepsies which becomes a limiting factor for a high population low‐resource setting like ours. The onset of seizures can be missed by the primary care giver; seizures with mild motor movements or non‐motor seizures and epileptic spasms are often missed. Ictal EEG is difficult to obtain leading to lapses in classification at level 1. However, there is considerable overlap between level I and level II (90%) in our study which does suggest a degree of redundancy and duplication in classification despite allowing accommodation of as many syndromes as possible. The 2017 ILAE classification improves upon the previous classification systems while incorporating development in recent years as it provides us the flexibility required to classify epilepsy with an incomplete knowledge of the disease pathophysiology and the limited resources in low‐ and middle‐income countries.

The limitations of the study are that it is a hospital‐based study and not a population‐based study. Therefore, the electroclinical spectrum may not be a true reflection of the population characteristics. As ours is a tertiary care center, there may be a relatively greater proportion of pharmacoresistant and difficult to diagnose cases. Also, ictal EEG was not performed in all cases. This is, however, consistent with usual epilepsy clinical practice where the large majority of patients undergo interictal EEG testing and ictal EEG tends to be performed commonly in patients undergoing VEEG testing. Additionally, in seizure classification, we have used the dominant seizure type. This may have led to an underestimation of the actual prevalence of each seizure type since children with complex epilepsies often have seizures of multiple types. There are very few studies on the applicability of the 2010 and 2017 ILAE classification of epilepsy and the electroclinical spectrum of epilepsy, more so in an exclusive pediatric population. Also, this study has a large sample size leading to a good coverage of the diverse spectrum of epilepsy.

## 5 CONCLUSION

This study showed that the classification systems proposed by ILAE in 1989 were statistically superior to the 2010 scheme although equivalent to the 2017 scheme in epilepsy classification. The 2010 and 2017 schemes were statistically equivalent. However, the 2010 and 2017 ILAE classification delineate the etiology of epilepsy with less ambiguity. New syndromes have been added with information congregated in the past three decades. However, some of the syndromes and the category of distinct constellations need more explanation. The 2017 classification allows for a multidimensional classification which allows classification in a resource‐limited setting and to add new information when acquired. Level I can be a complete descriptive entity of the seizure semiology while level II can incorporate the localization of the epileptogenic focus/type of epilepsy based on information gathered by EEG and MRI. Hence, we should consider the 2017 ILAE classification of epilepsy not as an absolute end to our endeavor to classify epilepsy scientifically but as a milestone in paving the way forward to reaching an ideal classification for epilepsy.

## CONFLICTS OF INTEREST

None. We confirm that we have read the Journal's position on issues involved in ethical publication and affirm that this report is consistent with those guidelines.
